# A Rare Case of Light Chain Amyloidosis of the Gastrointestinal Tract

**DOI:** 10.1155/2020/1921805

**Published:** 2020-06-30

**Authors:** Kathrin Dvir, Gliceida M. Galarza-Fortuna, Anna Willet, Christopher Febres-Aldana, Nathaly Cortez, Samuel Rapaka, Andre Coombs, Robert Goldberg, Michael Schwartz, Kfir Ben-David

**Affiliations:** ^1^Department of Internal Medicine, Mount Sinai Medical Center, USA; ^2^The Arkadi M. Rywlin M.D. Department of Pathology, Mount Sinai Medical Center, USA; ^3^Department of General Surgery, Mount Sinai Medical Center, USA; ^4^Department of Gastroenterology, Mount Sinai Medical Center, USA; ^5^Comprehensive Cancer Center, Mount Sinai Medical Center, USA

## Abstract

A 65-year-old Hispanic female presented with a one-year history of anorexia, nausea, early satiety, epigastric discomfort, and a 20 kg weight loss. Computed tomography (CT) demonstrated heterogeneous liver parenchyma. Upper endoscopy revealed large, fungating, infiltrative mass at the lesser gastric curvature incisura, highly suspicious of gastric tumor; however, initial biopsy of the gastric mass was equivocal and an exploratory laparoscopy was performed. Repeated intraoperative biopsies of the gastric mass and of liver parenchyma demonstrated diffuse hyalinized stroma consistent with amyloid deposition, and a bone marrow biopsy confirmed the diagnosis of primary light chain (AL) amyloidosis.

## 1. Background

Light chain (AL) amyloidosis is a plasma cell dyscrasia, characterized by the misfolding of extracellular proteins produced by an abnormal small plasma-cell clone that aggregate and deposit in tissues [1, 2]. It is defined as a rare disease, with an incidence of 1 case per 100,000 person-years in Western countries [1]. The most commonly involved organs are the heart (75%), kidneys (65%), liver (15%), soft tissues (15%), and peripheral and/or autonomic nervous system (10%). Macroglossia, although considered a hallmark feature of AL amyloidosis, presents in a minority of patients. AL amyloidosis of the gastrointestinal (GI) tract, with biopsy-proven disease, is uncommon, and found in 5% of cases [3, 4].

## 2. Case Presentation

**Figure 1:**
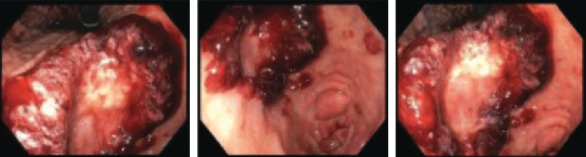
Upper endoscopy findings. Left to right: incisura of the stomach, gastric body, gastric body mass.

A 65-year-old Hispanic female with a history of Barrett's esophagus and a large gastric ulcer presented with a one-year history of loss of appetite, early satiety, epigastric discomfort, nausea, a 20 kg weight loss, and a petechial rash on her hands and face that waxed and waned. Although she denied blood in her stools or hematemesis, she was found to have microcytic hypochromic anemia with hemoglobin of 8.8 g/dL, hematocrit 29%, and MCV 79.5 fL. Iron studies confirmed iron deficiency of 22 mcg/dL (normal value 45-160 mcg/dL), low ferritin of 16 ng/mL (normal value 20-288 ng/mL), low iron saturation 5% (normal value 11-50%), and normal total iron binding capacity (TIBC). Aspartate transaminase (AST) and alanine aminotransferase (ALT) were within normal limit; however, alkaline phosphatase (ALP) was elevated at 314 U/L. Coagulation tests and renal function test were normal. A computed tomography of the abdomen and pelvis showed heterogeneous appearance of the liver parenchyma. The patient was started on intravenous iron transfusion with adequate response to treatment. Tumor markers (AFP, CA 19-9, CEA), IgG4, mitochondrial antibody, and ANA were negative. No pathological findings were identified on colonoscopy; however, a large fungating, infiltrative, and polypoid, noncircumferential mass with bleeding and stigmata of recent bleeding was found at the lesser curvature incisura with mild antral gastritis, highly suspicious of gastric carcinoma (Figure 1).

**Figure 2:**
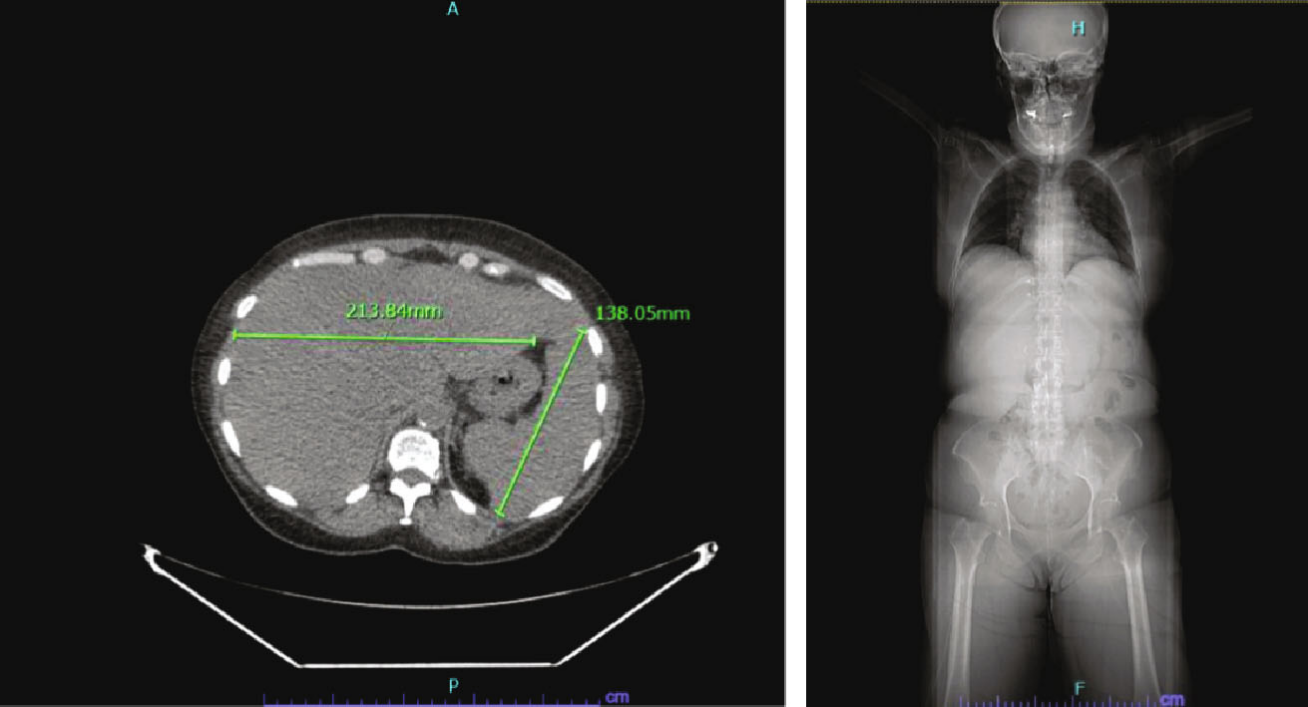
CT scan skull base to midthigh findings. Axial view (a): the spleen and liver borderline enlarged. Coronal view (b): no focal abnormalmality in the distal esophagus and stomach with no masses, adenopathy, or further evidence of regional or distal metastatic disease.

Biopsies did not confirm gastric malignancy. Positron emission tomography–computed tomography (PET/CT) scan of skull base to midthigh was performed showing no evidence of regional or distal metastatic disease. The scan was significant for small amount of fluid in the pelvis, anasarca, and mild hepatosplenomegaly (Figure 2).

The patient had an exploratory laparoscopy with repeat esophagogastroduodenoscopy and biopsies. On laparoscopy, she was noted to have a markedly enlarged left lobe of the liver which was biopsied.

**Figure 3:**
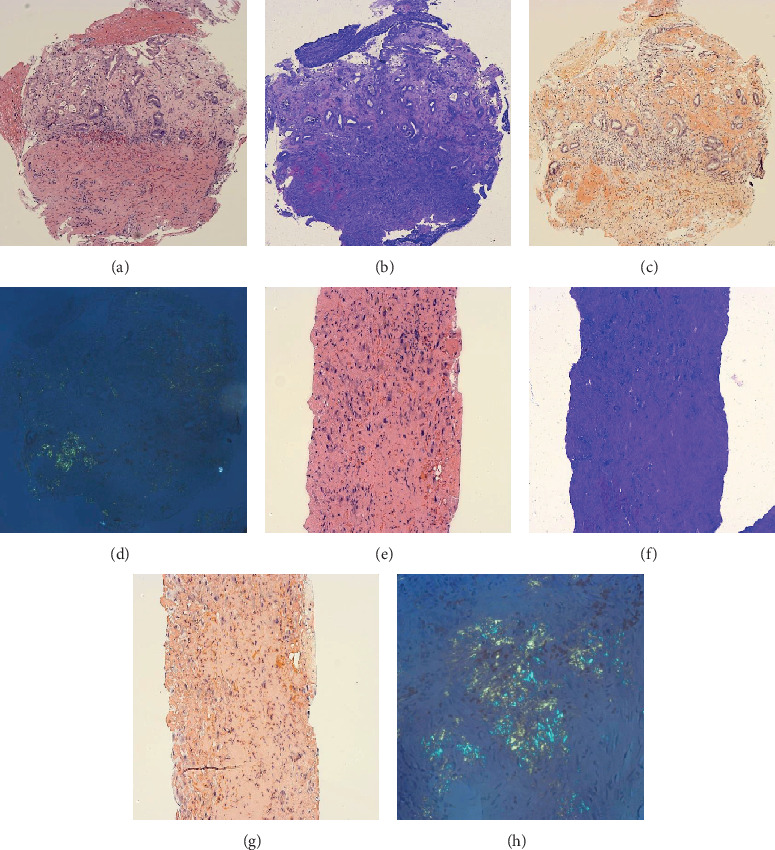
Amyloid deposition in the stomach and liver. (a–d) Gastric mucosa with ulceration, fibrinopurulent exudate, regenerative changes, and hyalinization of lamina propria secondary to amyloid deposition (pink on hematoxylin and eosin (H&E), (a)), highlighted by a metachromatic reaction on crystal violet stain (magenta, (b)) and congophilia on Congo red stain (red, (c)) with apple-green birefringence on polarized light (d). (e–h). Core liver biopsy with diffuse hyalinized stroma (H&E, (e)) consistent with amyloid deposition demonstrated by crystal violet (f), Congo red stain (g), and apple-green birefringence on polarized light (h). Magnification: (a–d), 100x; (e–g), 200x; (h), 400x.

Pathology of liver tissue revealed diffuse hyalinized stroma consistent with amyloid deposition. The gastric mucosa also showed focal hyalinization of the lamina propria consistent with amyloid deposition. Special stains for Congo red and crystal violet were positive, supporting the diagnosis (Figure 3).

Further work up to differentiate between systemic light chain (AL) amyloidosis from secondary amyloidosis (AA) was performed. Serum myeloma parameters showed normal kappa light chains at 10.4 mg/dL; however, elevated lambda light chain 494.9 mg/dL and the kappa/lambda ratio was low at 0.02 (normal range 0.26 to 1.65) pointing towards the diagnosis of light chain (AL) amyloidosis.

**Figure 4:**
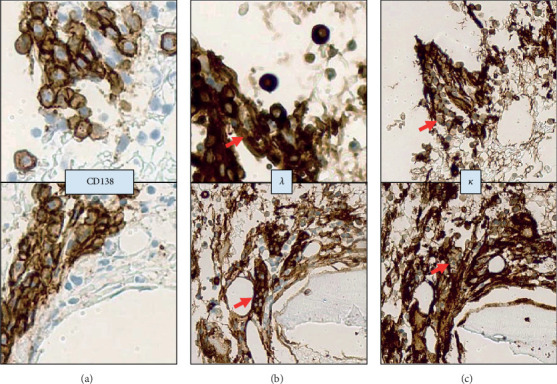
Bone marrow biopsy showing a plasma cell neoplasm with lambda light chain restriction. Clusters of plasma cells in perivascular spaces highlighted by CD-138 immunostaining (a) with strong expression of lambda light chain (*λ* (b)) and negativity for kappa light chain (*κ* (c)). All microphotographs shown at high magnification, 600x.

Finally, a bone marrow biopsy was performed confirming plasma cell neoplasm with lambda light chain restriction involving 14% of bone marrow cellularity. Excess free lambda light chains were consistent with diagnosis of primary systemic light chain amyloidosis (Figure 4).

## 3. Outcome

The patient was started on treatment with cyclophosphamide, bortezomib, and dexamethasone (CyBorD regimen). Pro-brain-natriuretic peptide was found to be mildly elevated at 181 pg/mL, and the patient was referred to cardiology. An echocardiogram resulted in severe restrictive cardiomyopathy, biventricular heart failure with reduced ejection fraction of 49%, highly suspicious of cardiac infiltration by AL amyloid. As a result of right heart failure, the patient developed substantial abdominal ascites and peripheral edema which was managed with scheduled paracenteses and diuresis. Hepatic vein and portal vein were confirmed to be patent via Doppler ultrasound. The patient was noted to have persistent nausea, dyspepsia, and early satiety which were attributed to gastric infiltration and dysmotility secondary to amyloidosis.

She was further evaluated for peripheral blood stem cell transplant but was not deemed a candidate. Liver transplant was not indicated in this case, given no significant liver compromise and the involvement of extrahepatic organs.

The patient continued on maintenance CyBorD regimen with improvement of all myeloma parameters to normal levels.

## 4. Discussion

The toxic monoclonal light-chain proteins in AL can damage virtually any organ, with the exception of the brain, and most frequently affect the heart and kidneys [4, 5]. Unfavorable outcomes in AL amyloidosis are classically associated with cardiac involvement, but GI involvement, although less frequent, is associated with an extensive disease with more organ involvement and a shorter overall survival [6]. Interestingly, in these patients, as in our patient, renal involvement is not commonly seen [7, 8].

AL amyloidosis is difficult to recognize because of its broad range of manifestations and what are often vague symptoms. AL amyloidosis of GI involvement often presents with early satiety, diarrhea, and weight loss. Hepatomegaly is common and can occur as a result of either congestion from right heart failure or amyloid infiltration of the liver [9]. Both of these findings were present in our patient.

The diagnosis of AL amyloidosis is challenging and requires (1) demonstration of amyloid on tissue biopsy, which often necessitates multiple biopsy sites, as was the case in our patient, and (2) demonstration of a plasma cell dyscrasia. Disease modifying treatment aiming to suppress the underlying plasma cell dyscrasia with high-dose chemotherapy followed by autologous stem-cell transplantation is the mainstay of management. Liver transplantation is an option for a select group of patients with progressive liver failure secondary to hepatic infiltration, in the absence of significant extrahepatic involvement [8, 9].

## Data Availability

No data were used to support this study.

## Additional Points

*Learning Point and Take Home Messages*. GI involvement in AL amyloidosis is uncommon but carries a high morbidity and confers poor prognosis. High level of suspicion should be kept when malignancy is clinically suggested amid equivocate biopsy results. Escalating diagnostic approach with repeat biopsy attempts in different organs should be employed in order to maximize diagnostic yield. Early diagnosis remains vital for improving treatment efficacy and overall survival. Multidisciplinary approach should be employed to reach prompt diagnosis and improve patients' outcomes. Awareness of GI AL amyloidosis should be enhanced amongst clinicians.
